# Preoperative lactate dehydrogenase-to-albumin ratio as a nutrition–inflammation index associated with postoperative pneumonia after McKeown esophagectomy: a retrospective cohort study

**DOI:** 10.3389/fnut.2026.1823767

**Published:** 2026-08-05

**Authors:** Chao Liu, Xiang Gao, Bo Feng, Dafu Xu, Derong Tang, Bao Zang, Zhiwei Xu, Jianqiang Zhao, Qingquan Wu, Jian Ji, Zhiyun Xu

**Affiliations:** 1 Department of Thoracic Surgery, The Affiliated Huai’an No. 1 People’s Hospital of Nanjing Medical University, Huai’an, China; 2 Department of Cardiothoracic Surgery, Jinling Hospital, Affiliated Hospital of Medical School, Nanjing University, Nanjing, China; 3 School of Clinical Medicine, Hunan University of Medicine, Huaihua, China

**Keywords:** esophageal cancer, lactate dehydrogenase-to-albumin ratio, McKeown esophagectomy, nutrition-inflammation index, perioperative risk stratification, postoperative pneumonia

## Abstract

**Background:**

Postoperative pneumonia is a frequent and clinically consequential complication after esophagectomy. The lactate dehydrogenase (LDH)-to-albumin ratio (LAR) integrates LDH-associated metabolic-inflammatory burden and albumin-associated nutritional-inflammatory reserve, but its association with postoperative pneumonia after McKeown esophagectomy remains unclear. This study evaluated whether preoperative LAR is associated with postoperative pneumonia in a clinically homogeneous, surgery-first cohort.

**Methods:**

We retrospectively analyzed 467 consecutive patients with esophageal cancer who underwent curative-intent McKeown esophagectomy without neoadjuvant therapy between January 2022 and December 2024. LAR was calculated from LDH and albumin levels measured within 7 days before surgery and was analyzed primarily as a continuous variable. ROC-derived binary LAR was retained as a supplementary sensitivity analysis. The primary endpoint was postoperative pneumonia within 30 days. Discrimination was assessed using ROC analysis and DeLong’s test. Associations were evaluated using staged multivariable logistic regression analyses, restricted cubic spline analyses, decision curve analyses, subgroup and timing analyses, and LDH–albumin joint-effect analyses.

**Results:**

Postoperative pneumonia occurred in 147 of 467 patients (31.5%). The median onset time was postoperative day (POD) 6 [interquartile range (IQR), 4–9], and 98 events (66.7%) occurred within the first postoperative week. LAR showed good discrimination for postoperative pneumonia (AUC = 0.803), with an optimal Youden index cutoff of 5.855. Continuous LAR remained independently associated with postoperative pneumonia after full adjustment [Model 3 odds ratio (OR) per one-unit increase, 2.568; 95% CI, 2.085–3.163; *p* < 0.001], and estimates were stable after additional adjustment for surgical approach and conversion to open surgery. Restricted cubic spline analysis showed a significant overall association and non-linearity (P-overall < 0.001; P-non-linearity = 0.004). Pneumonia incidence increased across RCS-derived LAR strata from 7.9 to 25.9 and 59.8%. Binary LAR, subgroup analyses, early- and later-onset pneumonia analyses, and LDH–albumin joint-effect analyses supported the robustness and biological plausibility of the association.

**Conclusion:**

Preoperative LAR was independently associated with postoperative pneumonia after McKeown esophagectomy when analyzed as a continuous nutrition-inflammation index. LAR may support perioperative risk stratification and early postoperative surveillance, but the internally derived threshold requires external validation before its clinical generalization.

## 1 Introduction

Esophageal cancer is a highly aggressive malignancy and remains a major cause of cancer-related morbidity and mortality worldwide (1), with esophageal squamous cell carcinoma being particularly common in East Asia (2). For patients with resectable disease, esophagectomy remains a central component of curative-intent treatment (3, 4). However, despite advances in minimally invasive techniques, anesthesia, perioperative care, and enhanced recovery pathways, esophagectomy is still associated with substantial postoperative morbidity (5, 6). Among postoperative complications, pneumonia is one of the most frequent and clinically consequential complications after esophagectomy (7, 8). It may lead to prolonged mechanical ventilation, delayed mobilization and oral intake, intensive care unit (ICU) readmission, longer hospitalization, and interrupted or delayed subsequent oncologic treatment (9, 10). Therefore, identifying patients who are vulnerable to postoperative pneumonia before clinical deterioration occurs remains an important perioperative challenge.

Postoperative pneumonia after esophagectomy is not determined by surgical exposure alone. Rather, it reflects a complex interaction between operative stress, perioperative pulmonary injury, aspiration risk, impaired airway clearance, systemic inflammation, and host reserve (7, 8). In this context, nutritional and inflammatory status are particularly relevant (11, 12). Malnutrition, hypoalbuminemia, systemic inflammatory activation, and catabolic stress may impair immune competence, delay tissue repair, aggravate endothelial dysfunction, and reduce tolerance to perioperative pulmonary insults (13, 14). Several clinical scores and inflammatory or nutrition-based biomarkers have been evaluated for postoperative complications after major surgery; however, many models require multiple variables, have undergone inconsistent validation, or may be difficult to integrate into routine preoperative workflows. A simple, reproducible, and routinely available biomarker reflecting both metabolic-inflammatory burden and nutritional-inflammatory reserve could therefore be useful for perioperative risk stratification.

Lactate dehydrogenase (LDH) and albumin represent two biologically complementary dimensions of perioperative vulnerability. LDH is a widely available laboratory marker associated with tissue injury, hypoxia-related metabolic stress, anaerobic glycolysis, tumor burden, and systemic inflammation (15). Elevated LDH may therefore reflect a state of increased metabolic and inflammatory burden before surgery. Albumin, in contrast, is not only a nutritional marker but also a negative acute-phase reactant and a surrogate marker of inflammation-related catabolism, vascular permeability, antioxidant capacity, and physiologic reserve (14, 16). Lower albumin levels have been associated with impaired host defense and postoperative infectious complications (14, 16). The lactate dehydrogenase-to-albumin ratio (LAR), by combining LDH and albumin, may capture a “high-burden/low-reserve” phenotype that is not fully represented by either component alone. This phenotype is biologically plausible in relation to postoperative pneumonia because patients entering surgery with greater metabolic-inflammatory stress and lower nutritional reserve may have a reduced ability to withstand one-lung ventilation, surgical trauma, aspiration risk, and early postoperative inflammatory responses.

Previous studies have mainly investigated LAR as a prognostic biomarker in malignancies, including gastrointestinal cancers and esophageal cancer (17, 18). Those studies support the concept that LAR reflects tumor–host interactions, systemic inflammation, metabolic stress, and nutritional status. However, long-term prognosis and short-term postoperative pneumonia represent clinically distinct endpoints. Prognosis is influenced by tumor biology, pathological stage, recurrence, adjuvant therapy, and long-term competing risks, whereas postoperative pneumonia is an early perioperative event more directly related to host vulnerability, pulmonary reserve, nutritional-inflammatory status, and surgical stress. Therefore, the prognostic relevance of LAR does not preclude its potential relevance for postoperative infectious morbidity. Instead, the same biological domains represented by the LAR may also be relevant to early postoperative pneumonia, particularly in a clinically homogeneous surgery-first cohort.

Accordingly, we investigated the association between preoperative LAR and postoperative pneumonia within 30 days after McKeown esophagectomy in patients with esophageal cancer who did not receive neoadjuvant therapy. We also evaluated the discriminative performance of LAR, compared LAR with its individual components, assessed the functional form of the LAR–pneumonia association, examined clinical utility using decision curve analysis, and described perioperative management characteristics and the timing distribution of postoperative pneumonia onset. We did not aim to establish LAR as a causal mediator or a stand-alone diagnostic test. Rather, we aimed to determine whether LAR could serve as a pragmatic nutrition–inflammation marker associated with postoperative pneumonia and potentially support individualized perioperative risk stratification, closer surveillance, and targeted pulmonary and nutritional optimization.

## 2 Methods

### 2.1 Study design, setting, and participants

**Figure 1:**
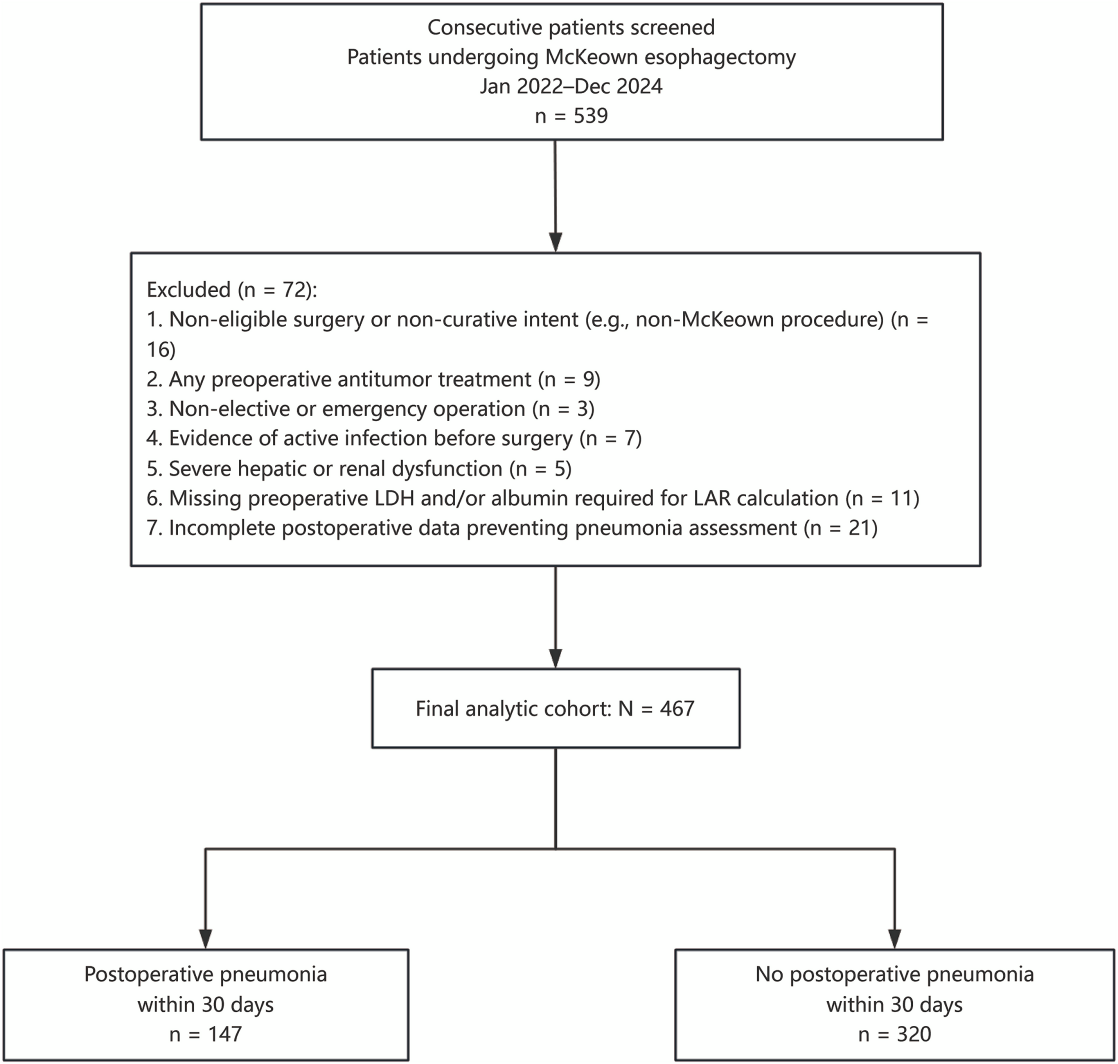
Patient selection flowchart. A total of 539 consecutive patients who underwent McKeown esophagectomy between January 2022 and December 2024 were screened. After excluding 72 patients according to predefined criteria, 467 patients were included in the final analytic cohort. Among them, 147 developed postoperative pneumonia within 30 days after surgery and 320 did not.

This retrospective observational cohort study included consecutive patients with esophageal cancer who underwent elective McKeown esophagectomy with curative intent at The Affiliated Huai’an No. 1 People’s Hospital of Nanjing Medical University between January 2022 and December 2024. The study was conducted and reported in accordance with the Strengthening the Reporting of Observational Studies in Epidemiology (STROBE) guidelines (19). Patients were included if they met all of the following criteria: (1) histologically confirmed esophageal cancer treated with curative-intent resection; (2) elective McKeown esophagectomy; (3) no preoperative antitumor therapy, including neoadjuvant chemotherapy, radiotherapy, immunotherapy, or targeted therapy; (4) availability of preoperative serum lactate dehydrogenase (LDH) and albumin values for calculation of the lactate dehydrogenase-to-albumin ratio (LAR); and (5) postoperative clinical records sufficient to diagnose postoperative pneumonia. Patients were excluded if they met any of the following criteria: non-eligible surgery or non-curative intent, receipt of any preoperative antitumor therapy, non-elective or emergency surgery, evidence of active infection before surgery, severe hepatic and/or renal dysfunction, missing LDH or albumin data precluding LAR calculation, or incomplete postoperative data preventing pneumonia assessment. A total of 539 consecutive patients were screened during the study period. After applying the predefined inclusion and exclusion criteria, 467 patients were included in the final analytic cohort. Patients were categorized according to whether postoperative pneumonia occurred within 30 days after surgery. The patient selection process is shown in Figure 1. The study protocol was reviewed and approved by the Ethics Committee of Nanjing Medical University (Approval No. KY-2026-072-01). Given the retrospective design, the use of existing de-identified records, and the absence of additional intervention or patient risk, the requirement for written informed consent was waived by the Ethics Committee. The study was conducted in accordance with the Declaration of Helsinki. Preoperative active infection was defined as a documented or clinically suspected infection within 7 days before surgery requiring antimicrobial treatment, accompanied by compatible symptoms or signs, microbiological evidence, or imaging findings, including active respiratory infection, urinary tract infection, abdominal infection, bloodstream infection, or another infection judged by the treating team to be clinically active before surgery. Isolated elevation of C-reactive protein (CRP), leukocyte count, or other inflammatory markers without clinical evidence of active infection was not considered sufficient for exclusion because subclinical inflammatory burden was part of the host phenotype under investigation.

### 2.2 Exposure definition: lactate dehydrogenase-to-albumin ratio

The primary exposure was preoperative LAR, which was calculated as LDH (U/L) divided by albumin (g/L). Preoperative LDH and albumin values were obtained within 7 days before surgery. When multiple measurements were available during this window, the value closest to the date of surgery was used. LAR was analyzed as a continuous variable in the primary association analyses presented in Table 1. For supplementary sensitivity analyses, LAR was dichotomized using the ROC-derived Youden index cutoff of 5.855. High LAR was defined as LAR ≥5.855, and low LAR was defined as LAR <5.855. This dichotomized threshold was used as a study-specific benchmark for interpretability and sensitivity analysis rather than as a universal clinical decision threshold.

**Table 1:** Multivariable logistic regression models evaluating preoperative LAR (continuous) for postoperative pneumonia.

Model	*n*	Events	OR (95% CI) for LAR (per 1-unit increase)	*P*-value
Crude	467	147	2.389 (1.976–2.890)	<0.001
Model 1	467	147	2.423 (1.998–2.939)	<0.001
Model 2	467	147	2.513 (2.056–3.072)	<0.001
Model 3	466	147	2.568 (2.085–3.163)	<0.001
Model 4	466	147	2.587 (2.098–3.191)	<0.001
Model 5	466	147	2.572 (2.086–3.171)	<0.001
Model 6	466	147	2.589 (2.097–3.196)	<0.001

Multivariable logistic regression models were fitted to evaluate the association between preoperative LAR (continuous) and postoperative pneumonia. ORs correspond to a 1-unit increase in LAR. Model 1 adjusted for age, sex, and BMI. Model 2 additionally adjusted for smoking status, alcohol consumption, hypertension, diabetes mellitus, coronary heart disease, ASA physical status, FEV1/FVC, and LVEF. Model 3 further adjusted for operative duration, single-lung ventilation time, intraoperative blood loss, recurrent laryngeal nerve injury, tumor location, tumor length, tumor differentiation, and pTNM stage. Model 4 added surgical approach to Model 3; Model 5 added conversion to open surgery to Model 3; Model 6 added both surgical approach and conversion to open surgery to Model 3.

### 2.3 Outcome definition and timing of postoperative pneumonia

The primary endpoint was postoperative pneumonia within 30 days after McKeown esophagectomy. Pneumonia was treated as a binary outcome. For patients with multiple suspected episodes, only the first episode was counted. Postoperative pneumonia was adjudicated using a prespecified definition requiring radiographic evidence and supportive clinical features, consistent with the Esophagectomy Complications Consensus Group framework and standardized surveillance principles (20). Radiographic evidence was defined as a new or progressive pulmonary infiltrate, consolidation, or opacity compatible with pneumonia on chest radiography or computed tomography, as documented in the official radiology report. When imaging findings were equivocal, such as “infiltrate versus atelectasis” or “pneumonia versus pulmonary edema,” cases were classified as pneumonia only when subsequent imaging showed persistence or progression rather than rapid resolution. A pneumonia event was diagnosed when radiographic criteria were met and the patient exhibited at least two of the following clinical features within the same clinical episode: (1) fever >38.0 °C or hypothermia <36.0 °C; (2) leukocytosis >10 × 10^9^/L or leukopenia <4 × 10^9^/L; (3) purulent sputum or increased respiratory secretions; (4) new or worsening cough, dyspnea, or tachypnea; (5) microbiological confirmation from respiratory specimens, including sputum, endotracheal aspirate, or bronchoalveolar lavage, when available; and/or (6) worsening gas exchange, such as new or increased oxygen requirement, decreased peripheral oxygen saturation, or escalation of ventilatory support. Microbiological confirmation was not mandatory for diagnosis and was considered supportive when obtained as part of routine care. Two trained reviewers independently screened electronic medical records for potential pneumonia events. Disagreements were resolved by consensus under the supervision of the research team. For timing analyses, the postoperative day of pneumonia onset was defined as the first day on which the prespecified diagnostic criteria for pneumonia were fulfilled. The day of surgery was defined as postoperative day 0. Pneumonia onset was summarized as the median and interquartile range and was further categorized into POD 1–3, POD 4–7, POD 8–14, and POD 15–30. Early postoperative pneumonia was defined as pneumonia occurring on or before POD 7, whereas later pneumonia was defined as pneumonia occurring from POD 8 to POD 30.

### 2.4 Surgical approach, perioperative management, and postoperative recovery variables

All patients underwent McKeown esophagectomy, consisting of thoracic esophagectomy, abdominal gastric conduit reconstruction, and cervical anastomosis. To characterize surgical invasiveness, the surgical approach was classified as minimally invasive McKeown esophagectomy, hybrid McKeown esophagectomy, or open McKeown esophagectomy. Minimally invasive McKeown esophagectomy was defined as thoracoscopic thoracic access combined with laparoscopic abdominal access. Hybrid McKeown esophagectomy was defined as a combination of one minimally invasive phase and one open phase. Open McKeown esophagectomy was defined as open thoracotomy combined with open laparotomy. Thoracic access was further categorized as thoracoscopic, open thoracotomy, or converted open thoracotomy. Abdominal access was categorized as laparoscopic, open laparotomy, or converted open laparotomy. Conversion to open surgery was recorded separately. Intraoperative variables included operative duration, single-lung ventilation time, intraoperative blood loss, and recurrent laryngeal nerve injury. These variables were used to characterize operative stress and were considered in multivariable adjustment. Perioperative management and postoperative recovery variables were extracted from electronic medical records to describe respiratory care, ICU management, nutritional support, rehabilitation, steroid exposure, and inflammatory response. Postoperative respiratory and ICU-related variables included extubation in the operating room or post-anesthesia care unit, time to extubation, prolonged mechanical ventilation for ≥24 h, reintubation, initial ICU stay, ICU readmission, and cumulative ICU stay. Initial ICU stay was defined as the duration from postoperative ICU admission to first ICU discharge. Cumulative ICU stay included all ICU days during the index postoperative hospitalization, including ICU readmissions. Nutritional support variables included enteral access route, use of parenteral nutrition, duration of supplemental parenteral nutrition among parenteral nutrition users, timing of enteral nutrition initiation, timing of oral liquid intake, and timing of soft diet initiation. Enteral access was categorized as jejunostomy tube, nasojejunal tube, oral intake only/no feeding tube, or no early enteral access. Enteral nutrition start was defined as the first postoperative day on which planned enteral feeding was initiated. Oral liquid start and soft diet start were defined as the first postoperative day on which oral liquid intake and soft diet intake, respectively, were documented. Respiratory rehabilitation and mobilization variables included the first documented day of respiratory rehabilitation, receipt of respiratory rehabilitation on POD 0–1, first ambulation day, and ambulation within POD 1. Respiratory rehabilitation included documented breathing exercises, cough training, airway clearance, sputum expectoration assistance, and respiratory physiotherapy when clinically indicated. Mobilization was initiated when clinically tolerated according to the patient’s postoperative recovery status. Steroid exposure was recorded to address the potential influence of perioperative corticosteroid use on inflammatory response and postoperative pulmonary outcomes. Steroid-related variables included dexamethasone for postoperative nausea and vomiting, prophylactic high-dose steroid use, therapeutic postoperative steroid use, and any steroid exposure before pneumonia onset or before POD 3. For patients who developed pneumonia, “any steroid before pneumonia/POD3” referred to steroid exposure before pneumonia onset; for patients without pneumonia, it referred to steroid exposure before POD 3. Inflammatory response variables included preoperative C-reactive protein (CRP); CRP on POD 1, POD 3, POD 5, and POD 7; peak CRP during POD 0–7; duration of systemic inflammatory response syndrome (SIRS) during POD 0–30; SIRS duration ≥48 h; and fever duration. SIRS was assessed using conventional clinical criteria based on postoperative vital signs and laboratory data. SIRS duration was defined as the number of postoperative days during which SIRS criteria were fulfilled. Fever duration was defined as the number of postoperative days with body temperature >38.0 °C. These postoperative management, recovery, and inflammatory variables were summarized descriptively to characterize the perioperative clinical course and to address potential differences in surgical invasiveness and postoperative care. Variables that could occur after pneumonia onset or could be influenced by pneumonia, such as reintubation, ICU readmission, cumulative ICU stay, postoperative CRP trajectory, SIRS duration, fever duration, parenteral nutrition use, and delayed oral intake, were not included as covariates in the primary preoperative association models to avoid adjustment for potential downstream consequences of the outcome.

### 2.5 Covariates and data collection

Data were extracted from electronic medical records by trained research personnel using standardized data collection procedures. Baseline demographic variables included age, sex, and body mass index. Lifestyle factors included smoking status and alcohol consumption. Comorbidities included hypertension, diabetes mellitus, and coronary heart disease. Preoperative physiological variables included the American Society of Anesthesiologists (ASA) physical status, pulmonary function measured by FEV1/FVC, and left ventricular ejection fraction (LVEF). Laboratory variables included preoperative albumin, LDH, LAR, and CRP. Tumor-related variables included tumor location, tumor length, tumor differentiation, pathological T category, pathological N category, and pathological TNM stage. Pathological staging was assigned according to the AJCC/UICC 8th edition staging system (21). Data extraction was cross-checked by trained personnel, and key variables were reviewed by the research team. Standardized quality-control procedures were implemented to reduce transcription errors, resolve inconsistencies, and verify clinically implausible values.

### 2.6 Statistical analysis

**Figure 2:**
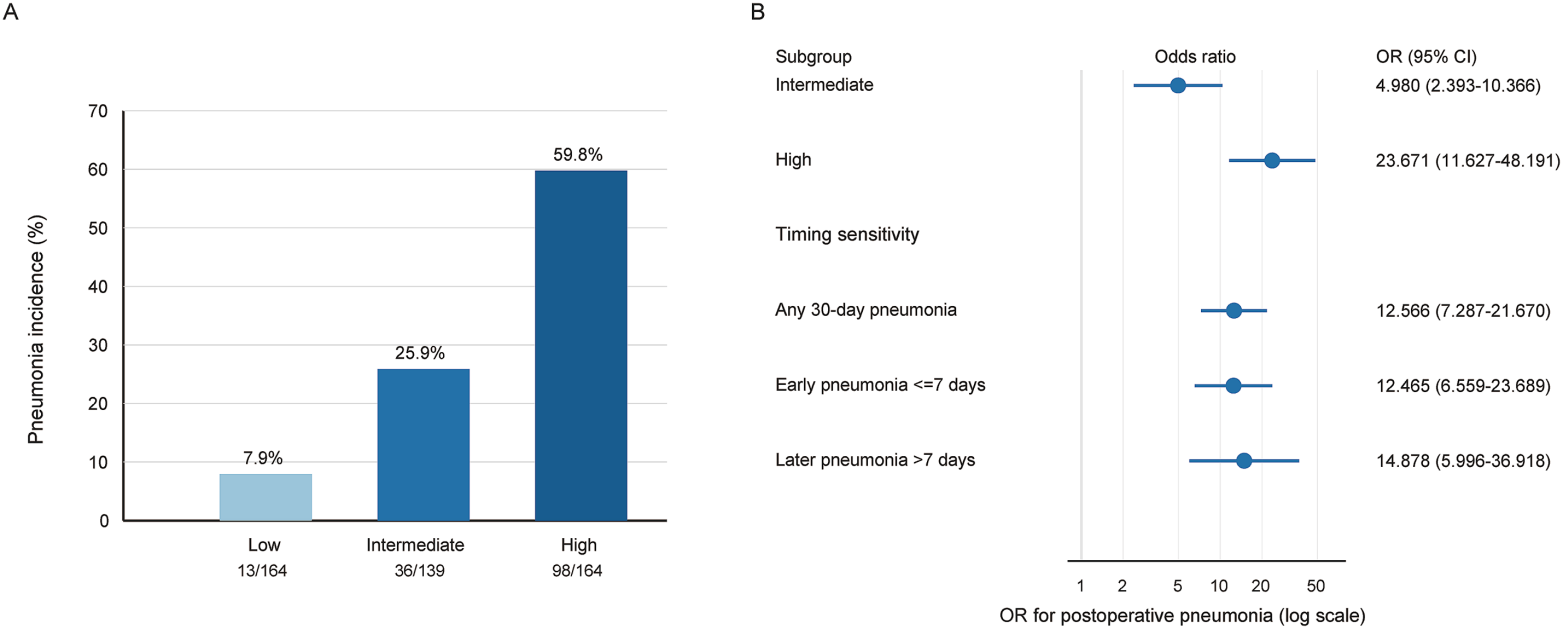
RCS-derived LAR risk stratification and timing sensitivity analyses. **(A)** Postoperative pneumonia incidence across RCS-derived LAR strata: low (<5.222), intermediate (5.222–6.424), and high (>6.424), with incidences of 7.9% (13/164), 25.9% (36/139), and 59.8% (98/164), respectively. **(B)** Adjusted odds ratios for the intermediate and high LAR strata compared with the low-risk reference stratum, together with timing sensitivity analyses for any 30-day postoperative pneumonia, early pneumonia on or before POD 7, and later pneumonia from POD 8 to POD 30. Detailed numerical results are provided in Supplementary Tables S6, S8.

**Figure 3:**
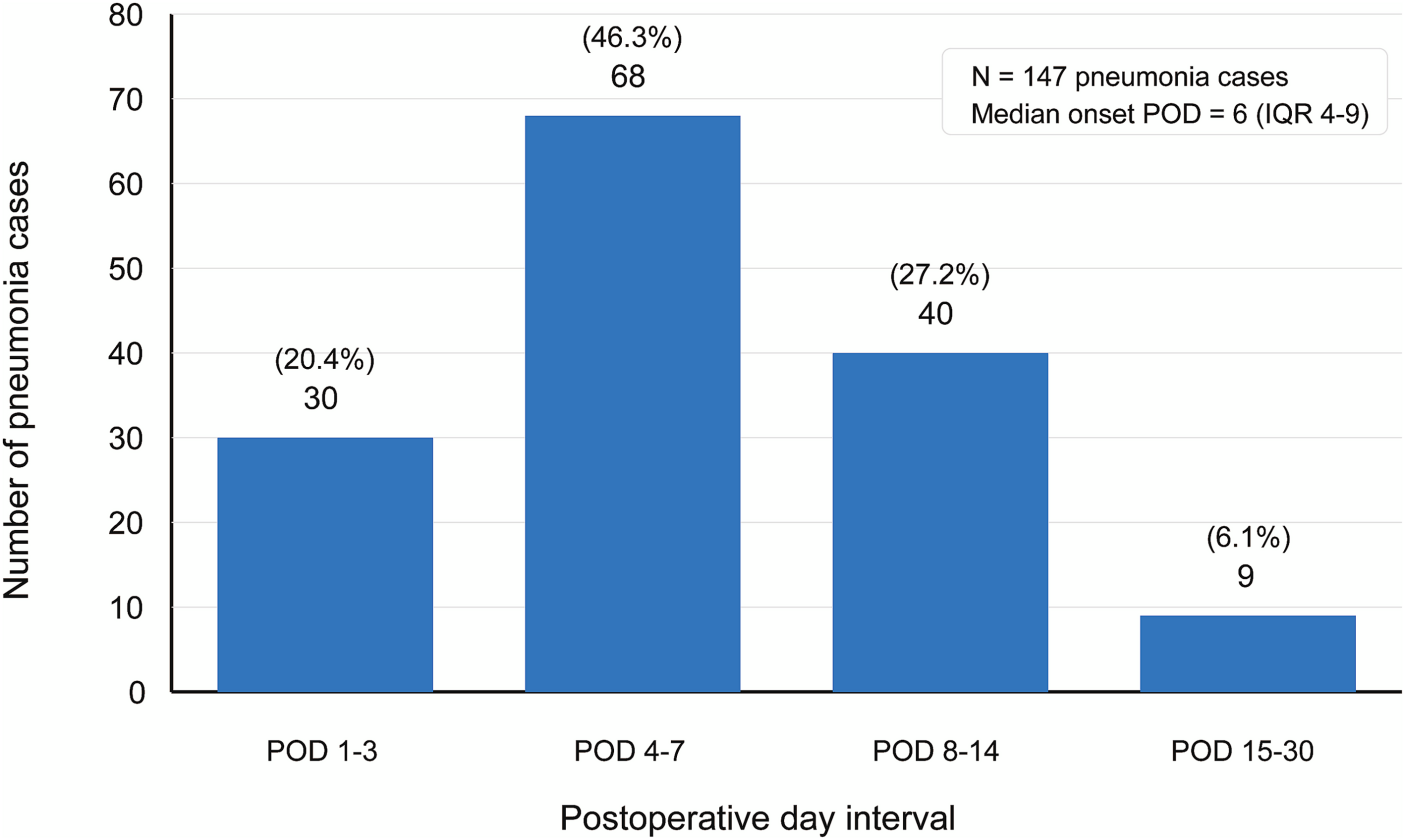
Timing distribution of postoperative pneumonia onset after McKeown esophagectomy. Among the 147 patients who developed postoperative pneumonia within 30 days after surgery, the median onset time was postoperative day 6 (IQR, 4–9). Pneumonia occurred most frequently during POD 4–7 (68/147, 46.3%), followed by POD 8–14 (40/147, 27.2%), POD 1–3 (30/147, 20.4%), and POD 15–30 (9/147, 6.1%). Overall, 98 of 147 pneumonia events (66.7%) occurred within the first postoperative week.

**Figure 4:**
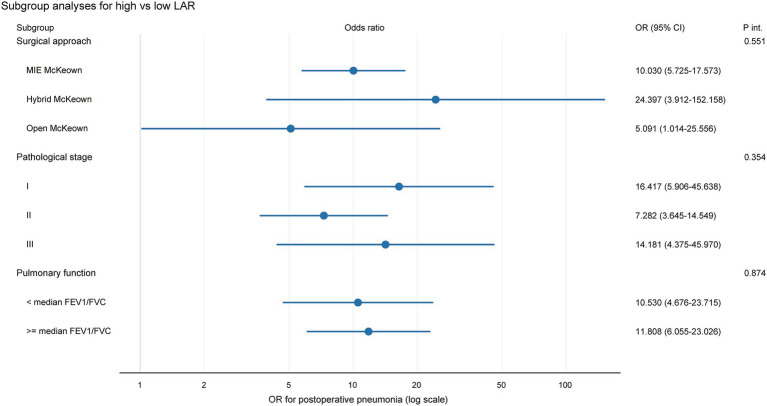
Subgroup forest plot for the association between high LAR and postoperative pneumonia. Odds ratios were estimated for ROC-derived high and low LARs within prespecified subgroups defined by surgical approach, pathological stage, and FEV1/FVC strata. Interaction tests did not show statistically significant effect modification by surgical approach (P-interaction = 0.551), pathological stage (P-interaction = 0.354), or FEV1/FVC strata (P-interaction = 0.874). Detailed subgroup estimates are provided in Supplementary Table S7.

**Figure 5:**
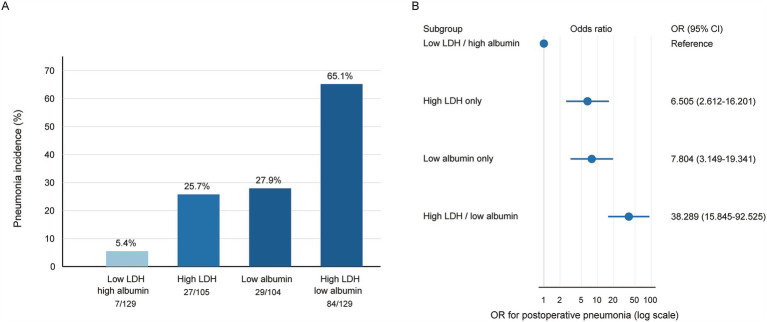
LDH–albumin joint-effect analysis for postoperative pneumonia. **(A)** Pneumonia incidence across joint categories of LDH and albumin. **(B)** Adjusted odds ratios for high LDH only, low albumin only, and combined high LDH/low albumin, using the low LDH/high albumin group as the reference. Patients with both high LDH and low albumin had the highest pneumonia incidence and the largest adjusted odds ratio. The analysis is exploratory and should be interpreted as joint risk identification rather than proof of causal synergy. Detailed numerical results are provided in Supplementary Table S9.

Baseline demographic, clinical, laboratory, and tumor characteristics were summarized for the overall cohort and stratified by postoperative pneumonia status, as shown in Table 2. Surgical approach, perioperative management, inflammatory response, and postoperative recovery variables are summarized in Table 3. Continuous variables with approximately normal distributions were presented as mean ± standard deviation and compared using Welch’s t test. Skewed continuous variables, including time-to-event, ICU, nutritional, rehabilitation, and inflammatory variables, were presented as median and interquartile range and compared using the Mann–Whitney U test. Categorical variables were presented as counts and percentages and compared using the chi-squared test or Fisher’s exact test, as appropriate. Absolute standardized mean differences were calculated to quantify between-group imbalance. The discrimination of preoperative LAR for postoperative pneumonia was evaluated using ROC analysis, and the area under the curve (AUC) was reported. The optimal cutoff value was determined using the Youden index. ROC curves for LAR, albumin, and LDH were compared using DeLong’s test (22). For ROC comparison, albumin was directionally aligned such that lower albumin corresponded to higher pneumonia risk. Univariable logistic regression analyses were performed for candidate baseline, laboratory, surgical, and tumor-related variables and are presented in Supplementary Table S1. The associations between LAR and postoperative pneumonia were evaluated using multivariable logistic regression models with staged covariate adjustment. LAR was modeled as a continuous variable in the primary analyses and is reported in Table 1. The crude model included LAR only. Model 1 was adjusted for age, sex, and body mass index (BMI). Model 2 was additionally adjusted for smoking status, alcohol consumption, hypertension, diabetes mellitus, coronary heart disease, ASA physical status, FEV1/FVC, and LVEF. Model 3 was further adjusted for operative duration, one-lung ventilation time, intraoperative blood loss, recurrent laryngeal nerve injury, tumor location, tumor length, tumor differentiation, and pTNM stage. To address the potential influence of surgical approach and conversion to open surgery, additional sensitivity models were fitted: Model 4 added surgical approach to Model 3; Model 5 added conversion to open surgery to Model 3; and Model 6 added both surgical approach and conversion to open surgery to Model 3. Binary LAR analyses using the ROC-derived cutoff were retained as supplementary sensitivity analyses and are reported in Supplementary Table S2. Restricted cubic spline analysis was performed to evaluate the functional form of the association between continuous LAR and postoperative pneumonia. Knots were placed at the 5th, 35th, 65th, and 95th percentiles of the LAR distribution, and the x-axis was restricted to the 5th–95th percentile range to reduce the influence of extreme values. The ROC-derived cutoff of 5.855 was used as the reference value. The overall association and non-linearity were assessed using likelihood-ratio tests. RCS-derived LAR risk strata were summarized in Supplementary Table S6 and are presented in Figure 2. Decision curve analysis was used to evaluate the clinical utility of the continuous LAR-based adjusted model (23). Predicted probabilities were derived from fully adjusted Model 3, and net benefit was calculated across threshold probabilities and compared with treat-all and treat-none strategies. The timing distribution of postoperative pneumonia onset is summarized in Figure 3 and Supplementary Table S3. Early postoperative pneumonia was defined as pneumonia occurring on or before POD 7, whereas later pneumonia was defined as pneumonia occurring from POD 8 to POD 30; these analyses are reported in Supplementary Table S8 and Figure 2. Postoperative outcomes and inflammatory response variables according to the LAR category are summarized descriptively in Supplementary Table S4. Prespecified subgroup analyses were performed according to surgical approach, pathological stage, and FEV1/FVC strata and are reported in Supplementary Table S7 and Figure 4. LDH–albumin interaction and joint-effect analyses were performed to explore whether the two components of LAR jointly characterized pneumonia risk; these exploratory analyses are reported in Supplementary Table S9 and Figure 5. Complete-case analysis was used for regression models. LDH, albumin, LAR, and pneumonia outcome data were complete for all 467 patients. Tumor length was missing in one patient, resulting in 466 complete cases in Model 3 and related adjusted analyses. The enteral nutrition start day was missing in 10 patients and was summarized descriptively. Pneumonia onset day and early pneumonia indicators were structurally applicable only among patients who developed pneumonia. A detailed missing-data summary is provided in Supplementary Table S5. No imputation was performed. All statistical tests were two-sided, and a *p* < 0.05 was considered statistically significant. Analyses and figures were generated using Python with pandas, NumPy, statsmodels, scikit-learn, and matplotlib.

**Table 2:** Baseline demographic, clinical, laboratory, and tumor characteristics of the study cohort stratified by postoperative pneumonia status.

Characteristic	Overall | (*n* = 467)	Postoperative pneumonia		*p-*value	|SMD|
		No | (*n* = 320)	Yes | (*n* = 147)		
Age (years)	65.39 ± 6.63	65.41 ± 6.70	65.32 ± 6.51	0.890	0.014
Sex				0.210	0.125
Female	212 (45.4%)	139 (43.4%)	73 (49.7%)		
Male	255 (54.6%)	181 (56.6%)	74 (50.3%)		
BMI (kg/m^2^)	22.64 ± 2.35	22.65 ± 2.32	22.61 ± 2.43	0.866	0.017
Smoking status				0.298	0.104
No	308 (66.0%)	216 (67.5%)	92 (62.6%)		
Yes	159 (34.0%)	104 (32.5%)	55 (37.4%)		
Alcohol consumption				0.558	0.058
No	320 (68.5%)	222 (69.4%)	98 (66.7%)		
Yes	147 (31.5%)	98 (30.6%)	49 (33.3%)		
Hypertension				0.702	0.038
No	238 (51.0%)	165 (51.6%)	73 (49.7%)		
Yes	229 (49.0%)	155 (48.4%)	74 (50.3%)		
Diabetes mellitus				0.995	0.001
No	394 (84.4%)	270 (84.4%)	124 (84.4%)		
Yes	73 (15.6%)	50 (15.6%)	23 (15.6%)		
Coronary heart disease				0.481	0.072
No	303 (64.9%)	211 (65.9%)	92 (62.6%)		
Yes	164 (35.1%)	109 (34.1%)	55 (37.4%)		
ASA physical status				0.643	0.094
I	139 (29.8%)	99 (30.9%)	40 (27.2%)		
II	315 (67.5%)	213 (66.6%)	102 (69.4%)		
III	13 (2.8%)	8 (2.5%)	5 (3.4%)		
FEV1/FVC (%)	102.70 ± 12.02	102.12 ± 12.36	103.97 ± 11.19	0.111	0.156
LVEF (%)	65.95 ± 2.28	65.88 ± 2.35	66.11 ± 2.11	0.305	0.101
Albumin (g/L)	38.71 ± 4.15	39.91 ± 3.78	36.09 ± 3.72	<0.001	1.018
LDH (U/L)	224.91 ± 45.29	214.43 ± 44.42	247.71 ± 38.36	<0.001	0.802
LAR	5.90 ± 1.46	5.43 ± 1.30	6.93 ± 1.28	<0.001	1.164
Tumor location				0.067	0.232
Upper	116 (24.8%)	83 (25.9%)	33 (22.4%)		
Middle	228 (48.8%)	163 (50.9%)	65 (44.2%)		
Lower	123 (26.3%)	74 (23.1%)	49 (33.3%)		
Tumor length (cm)	3.82 ± 0.69	3.84 ± 0.69	3.78 ± 0.69	0.388	0.086
Tumor differentiation				0.545	0.110
Well	27 (5.8%)	18 (5.6%)	9 (6.1%)		
Moderate	192 (41.1%)	137 (42.8%)	55 (37.4%)		
Poor	248 (53.1%)	165 (51.6%)	83 (56.5%)		
pT category				0.963	0.027
T1	163 (34.9%)	113 (35.3%)	50 (34.0%)		
T2	210 (45.0%)	143 (44.7%)	67 (45.6%)		
T3	94 (20.1%)	64 (20.0%)	30 (20.4%)		
pN category				0.984	0.002
N0	210 (45.0%)	144 (45.0%)	66 (44.9%)		
N1	257 (55.0%)	176 (55.0%)	81 (55.1%)		
pTNM stage				0.881	0.051
I	160 (34.3%)	112 (35.0%)	48 (32.7%)		
II	213 (45.6%)	144 (45.0%)	69 (46.9%)		
III	94 (20.1%)	64 (20.0%)	30 (20.4%)		

Continuous variables are presented as mean ± standard deviation and compared using Welch’s *t*-test. Categorical variables are presented as n (%) and compared using the χ^2^ test or Fisher’s exact test, as appropriate. Absolute standardized mean differences (|SMD|) were calculated to quantify between-group imbalance. Tumor length was summarized among available cases (overall *n* = 466; no-pneumonia group *n* = 319; pneumonia group *n* = 147).

**Table 3:** Surgical approach, perioperative management, inflammatory response, and postoperative recovery characteristics of the study cohort stratified by postoperative pneumonia status.

Characteristic	Overall | (*n* = 467)	Postoperative pneumonia		*P-*value	|SMD|
		No | (*n* = 320)	Yes | (*n* = 147)		
Surgical approach and intraoperative characteristics					
Surgical approach				0.677	0.088
MIE McKeown	371 (79.4%)	257 (80.3%)	114 (77.6%)		
Hybrid McKeown	56 (12.0%)	38 (11.9%)	18 (12.2%)		
Open McKeown	40 (8.6%)	25 (7.8%)	15 (10.2%)		
Thoracic access				0.157	0.192
Thoracoscopic	403 (86.3%)	279 (87.2%)	124 (84.4%)		
Open thoracotomy	60 (12.8%)	40 (12.5%)	20 (13.6%)		
Converted open thoracotomy	4 (0.9%)	1 (0.3%)	3 (2.0%)		
Abdominal access				0.418	0.132
Laparoscopic	385 (82.4%)	267 (83.4%)	118 (80.3%)		
Open laparotomy	76 (16.3%)	48 (15.0%)	28 (19.0%)		
Converted open laparotomy	6 (1.3%)	5 (1.6%)	1 (0.7%)		
Conversion to open, yes	14 (3.0%)	8 (2.5%)	6 (4.1%)	0.386	0.093
Operative duration (min)	275.73 ± 42.34	272.35 ± 40.50	283.09 ± 45.35	0.015	0.25
Single-lung ventilation time (min)	151.02 ± 44.15	150.73 ± 45.67	151.66 ± 40.76	0.826	0.021
Intraoperative blood loss (mL)	124.36 ± 55.02	122.43 ± 52.86	128.57 ± 59.42	0.285	0.109
Recurrent laryngeal nerve injury, yes	27 (5.8%)	21 (6.6%)	6 (4.1%)	0.286	0.106
Postoperative respiratory and ICU management					
Extubated in OR/PACU, yes	283 (60.6%)	198 (61.9%)	85 (57.8%)	0.405	0.083
Time to extubation (hours)	3.2 (1.8–13.7)	3.0 (1.8–12.5)	3.4 (1.8–16.6)	0.122	0.235
Prolonged ventilation ≥ 24 h, yes	37 (7.9%)	18 (5.6%)	19 (12.9%)	0.007	0.27
Reintubation, yes	17 (3.6%)	4 (1.2%)	13 (8.8%)	<0.001	0.405
Initial ICU stay (days)	1.0 (1.0–2.0)	1.0 (1.0–2.0)	1.0 (1.0–2.0)	0.349	0.095
ICU readmission, yes	27 (5.8%)	4 (1.2%)	23 (15.6%)	<0.001	0.617
Cumulative ICU stay (days)	1.0 (1.0–2.0)	1.0 (1.0–2.0)	1.0 (1.0–2.0)	<0.001	0.398
Nutritional support and postoperative diet					
Enteral access				0.186	0.219
Jejunostomy tube	334 (71.5%)	232 (72.5%)	102 (69.4%)		
Nasojejunal tube	100 (21.4%)	70 (21.9%)	30 (20.4%)		
Oral only/no feeding tube	23 (4.9%)	11 (3.4%)	12 (8.2%)		
No early enteral access	10 (2.1%)	7 (2.2%)	3 (2.0%)		
Parenteral nutrition, yes	63 (13.5%)	25 (7.8%)	38 (25.9%)	<0.001	0.528
Supplemental PN duration among PN users (days)	6.0 (5.0–8.0)	6.0 (5.0–8.0)	7.0 (5.2–8.0)	0.215	0.291
Enteral nutrition start (POD)	1.0 (1.0–1.0)	1.0 (1.0–2.0)	1.0 (1.0–1.0)	<0.001	0.39
Oral liquid start (POD)	7.0 (7.0–9.0)	7.0 (6.0–8.0)	9.0 (8.0–11.0)	<0.001	1.124
Soft diet start (POD)	11.0 (10.0–13.0)	11.0 (9.0–12.0)	13.0 (12.0–15.0)	<0.001	0.909
Respiratory rehabilitation and mobilization					
Respiratory rehabilitation start (POD)	1.0 (0.0–1.0)	1.0 (0.0–1.0)	1.0 (1.0–1.0)	0.021	0.235
Respiratory rehabilitation on POD0-1, yes	421 (90.1%)	294 (91.9%)	127 (86.4%)	0.065	0.184
First ambulation (POD)	1.0 (1.0–2.0)	1.0 (1.0–2.0)	2.0 (1.0–2.0)	0.008	0.26
Ambulation within POD1, yes	253 (54.2%)	183 (57.2%)	70 (47.6%)	0.054	0.192
Perioperative steroid exposure					
Dexamethasone for PONV, yes	235 (50.3%)	166 (51.9%)	69 (46.9%)	0.322	0.099
Prophylactic high-dose steroid, yes	12 (2.6%)	8 (2.5%)	4 (2.7%)	1.000	0.014
Therapeutic postoperative steroid, yes	31 (6.6%)	12 (3.8%)	19 (12.9%)	<0.001	0.369
Any steroid before pneumonia/POD3, yes	239 (51.2%)	169 (52.8%)	70 (47.6%)	0.297	0.104
Perioperative inflammatory response					
Preoperative CRP (mg/L)	4.7 (3.5–6.3)	4.5 (3.4–5.8)	5.5 (3.8–7.2)	<0.001	0.504
CRP on POD1 (mg/L)	87.1 (68.8–102.8)	87.2 (69.3–102.7)	86.8 (68.5–103.7)	0.792	0.03
CRP on POD3 (mg/L)	130.3 (110.8–153.4)	124.6 (106.3–144.2)	151.4 (122.1–172.2)	<0.001	0.689
CRP on POD5 (mg/L)	90.1 (69.2–120.0)	78.2 (61.9–98.5)	126.3 (102.8–151.3)	<0.001	1.334
CRP on POD7 (mg/L)	63.3 (41.6–89.2)	52.0 (37.8–69.7)	98.7 (71.2–126.3)	<0.001	1.231
Peak CRP on POD0-7 (mg/L)	137.6 (118.2–159.5)	127.7 (113.2–146.3)	160.4 (138.5–179.6)	<0.001	1.078
SIRS duration on POD0-30 (days)	1.0 (0.0–2.0)	1.0 (0.0–1.0)	2.0 (2.0–3.0)	<0.001	1.29
SIRS ≥ 48 h, yes	196 (42.0%)	75 (23.4%)	121 (82.3%)	<0.001	1.193
Fever duration (days)	1.0 (0.0–2.0)	1.0 (0.0–2.0)	3.0 (1.0–4.0)	<0.001	1.227

Data are presented as mean ± standard deviation, median (interquartile range), or n (%), as appropriate. Normally distributed intraoperative continuous variables were compared using Welch’s *t*-test; skewed time-to-event, ICU, nutritional, rehabilitation, and inflammatory variables were compared using the Mann–Whitney U test. Categorical variables were compared using the chi-square test or Fisher’s exact test, as appropriate. Absolute standardized mean differences (|SMD|) were calculated to quantify between-group imbalance. Supplemental PN duration was summarized among patients who received parenteral nutrition (overall *n* = 63; no-pneumonia *n* = 25; pneumonia *n* = 38). Enteral nutrition start was summarized among patients with available early enteral nutrition data (overall *n* = 457; no-pneumonia *n* = 313; pneumonia *n* = 144).

## 3 Results

### 3.1 Patient selection and incidence of postoperative pneumonia

A total of 539 consecutive patients undergoing McKeown esophagectomy between January 2022 and December 2024 were screened for eligibility. After applying the predefined inclusion and exclusion criteria, 467 patients were included in the final analytic cohort (Figure 1). Among them, 147 patients developed postoperative pneumonia within 30 days after surgery, corresponding to an overall incidence of 31.5%. The remaining 320 patients did not develop postoperative pneumonia during the 30-day postoperative observation period.

### 3.2 Baseline demographic, clinical, laboratory, and tumor characteristics

Baseline demographic, clinical, laboratory, and tumor characteristics stratified by postoperative pneumonia status are shown in Table 2. The overall cohort had a mean age of 65.39 ± 6.63 years and a mean BMI of 22.64 ± 2.35 kg/m^2^. Baseline demographic variables, lifestyle factors, comorbidities, ASA physical status, FEV1/FVC, and LVEF were generally comparable between patients with postoperative pneumonia and those without postoperative pneumonia, with small absolute standardized mean differences for the majority of variables. Preoperative laboratory profiles differed substantially between the groups. Compared with patients without postoperative pneumonia, those who developed postoperative pneumonia had lower albumin levels (36.09 ± 3.72 vs. 39.91 ± 3.78 g/L; *p* < 0.001; |SMD| = 1.018) and higher LDH levels (247.71 ± 38.36 vs. 214.43 ± 44.42 U/L; *p* < 0.001; |SMD| = 0.802). Consequently, LAR was significantly higher in the pneumonia group than in the non-pneumonia group (6.93 ± 1.28 vs. 5.43 ± 1.30; *p* < 0.001; |SMD| = 1.164). Tumor-related characteristics were broadly balanced between the groups. Tumor location, tumor length, tumor differentiation, pathological T category, pathological N category, and pathological TNM stage did not differ significantly between patients with postoperative pneumonia and those without postoperative pneumonia. In the overall cohort, pathological TNM stage was distributed as stage I in 160 patients (34.3%), stage II in 213 patients (45.6%), and stage III in 94 patients (20.1%).

### 3.3 Surgical approach, perioperative management, inflammatory response, and postoperative recovery

Surgical approach, perioperative management, inflammatory response, and postoperative recovery characteristics are summarized in Table 3. The majority of patients underwent minimally invasive McKeown esophagectomy (371/467, 79.4%), followed by hybrid McKeown esophagectomy (56/467, 12.0%) and open McKeown esophagectomy (40/467, 8.6%). The distribution of surgical approach did not differ significantly between patients with postoperative pneumonia and those without postoperative pneumonia (*p* = 0.677; |SMD| = 0.088). Thoracic access, abdominal access, and conversion to open surgery were also broadly comparable between the groups. Overall, thoracoscopic access was used in 403 patients (86.3%), laparoscopic abdominal access in 385 patients (82.4%), and conversion to open surgery occurred in 14 patients (3.0%). Operative duration was longer in the pneumonia group, whereas one-lung ventilation time, intraoperative blood loss, and recurrent laryngeal nerve injury were similar between the groups. Postoperative respiratory and recovery variables differed more clearly by pneumonia status. Patients who developed postoperative pneumonia more frequently required prolonged mechanical ventilation, reintubation, ICU readmission, and parenteral nutrition and showed delayed initiation of oral liquids and soft diets. Respiratory rehabilitation was generally initiated early in both groups, although first ambulation occurred later among patients who developed pneumonia. Dexamethasone use for postoperative nausea and vomiting, prophylactic high-dose steroid use, and any steroid exposure before pneumonia onset or before POD 3 did not differ significantly between the groups, whereas therapeutic postoperative steroid use was more frequent in patients who developed pneumonia. Inflammatory trajectories were also distinct. Patients with postoperative pneumonia had higher preoperative CRP and higher CRP levels on POD 3, POD 5, and POD 7, together with higher peak CRP during POD 0–7, longer SIRS duration, more frequent SIRS lasting ≥48 h, and longer fever duration. These perioperative and postoperative variables were summarized descriptively to characterize the clinical course and were not included as covariates in the primary preoperative association models when they could occur after, or be influenced by, pneumonia onset.

### 3.4 Timing distribution of postoperative pneumonia onset

The timing distribution of postoperative pneumonia onset is shown in Figure 3 and Supplementary Table S3. Among the 147 patients who developed postoperative pneumonia, the median onset time was POD 6 (IQR, 4–9). Pneumonia occurred most frequently during POD 4–7 (68/147, 46.3%), followed by POD 8–14 (40/147, 27.2%), POD 1–3 (30/147, 20.4%), and POD 15–30 (9/147, 6.1%). Overall, 98 of 147 pneumonia events (66.7%) occurred within the first postoperative week.

### 3.5 Discrimination of LAR and cutoff determination

**Figure 6:**
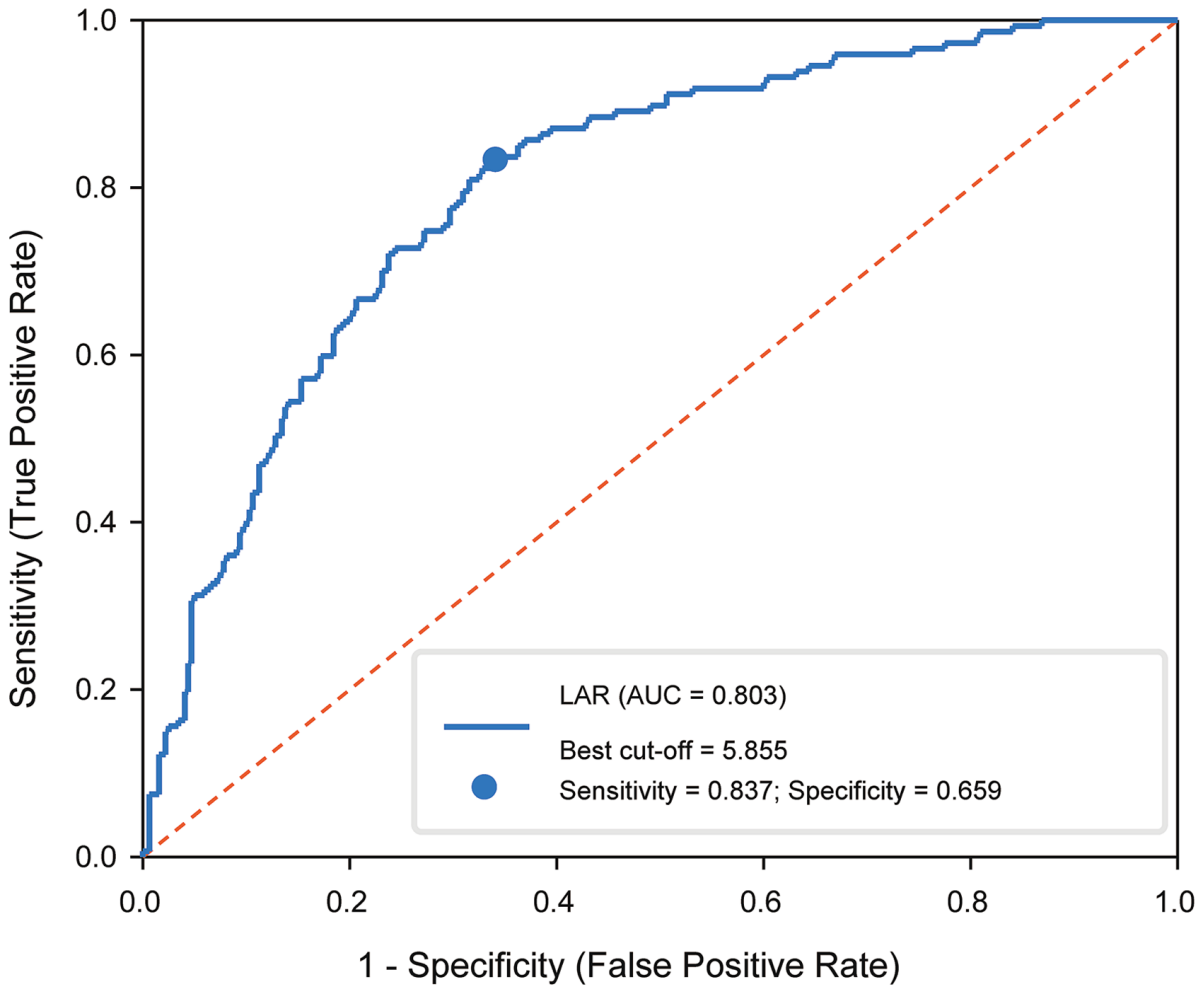
Receiver operating characteristic curve of preoperative LAR for postoperative pneumonia. The ROC curve shows the discriminative performance of preoperative LAR in relation to postoperative pneumonia after McKeown esophagectomy. The area under the curve was 0.803. The optimal cutoff value, determined using the Youden index, was 5.855, corresponding to a sensitivity of 0.837 and a specificity of 0.659.

The discriminative performance of preoperative LAR for postoperative pneumonia was evaluated using ROC analysis (Figure 6). LAR showed good discrimination, with an area under the curve (AUC) of 0.803. The optimal cutoff value determined by the Youden index was 5.855, corresponding to a sensitivity of 0.837 and a specificity of 0.659. This threshold was subsequently used to define high- and low-LAR categories for sensitivity analyses and descriptive stratification.

### 3.6 Comparative discrimination of LAR, albumin, and LDH

**Figure 7:**
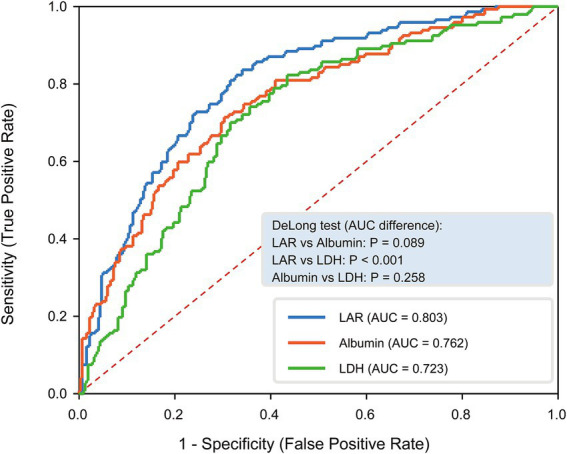
Comparative ROC curves of LAR, albumin, and LDH for postoperative pneumonia. ROC curves are shown for preoperative LAR, serum albumin, and LDH in terms of postoperative pneumonia. LAR had the highest area under the curve (AUC = 0.803), followed by albumin (AUC = 0.762) and LDH (AUC = 0.723). Pairwise comparisons were performed using DeLong’s test. LAR showed significantly better discrimination than LDH (*p* < 0.001), whereas the difference between LAR and albumin did not reach statistical significance (*p* = 0.089). The difference between albumin and LDH was not statistically significant (*p* = 0.258). Albumin was directionally aligned so that lower albumin corresponded to higher pneumonia risk.

ROC curves comparing LAR, albumin, and LDH are shown in Figure 7. LAR had the highest AUC among the three laboratory indicators (AUC = 0.803), followed by albumin (AUC = 0.762) and LDH (AUC = 0.723). Pairwise comparisons using DeLong’s test showed that LAR discriminated postoperative pneumonia significantly better than LDH (*p* < 0.001). The difference between LAR and albumin did not reach statistical significance (*p* = 0.089), and the difference between albumin and LDH was not statistically significant (*p* = 0.258). Therefore, LAR was interpreted as a complementary integrated nutrition–inflammation index rather than as a statistically proven replacement for albumin alone.

### 3.7 Univariable associations with postoperative pneumonia

Univariable logistic regression analyses are presented in Supplementary Table S1. Among laboratory variables, higher LAR was significantly associated with postoperative pneumonia (*p* < 0.001). Lower albumin and higher LDH were also significantly associated with pneumonia. Among surgical variables, operative duration showed a significant univariable association with postoperative pneumonia, whereas surgical approach, conversion to open surgery, single-lung ventilation time, intraoperative blood loss, and recurrent laryngeal nerve injury were not significantly associated with pneumonia in univariable analyses. The majority of baseline demographic, comorbidity, physiological, and tumor-related variables did not show statistically significant univariable associations with postoperative pneumonia.

### 3.8 Multivariable association between continuous LAR and postoperative pneumonia

The association between continuous preoperative LAR and postoperative pneumonia remained statistically significant after progressive covariate adjustment (Table 1). In the crude model, each one-unit increase in LAR was associated with higher odds of postoperative pneumonia (OR, 2.389; 95% CI, 1.976–2.890; *p* < 0.001). The association remained robust after adjustment for age, sex, and BMI in Model 1 (OR, 2.423; 95% CI, 1.998–2.939; *p* < 0.001), after additional adjustment for lifestyle factors, comorbidities, ASA physical status, FEV1/FVC, and LVEF in Model 2 (OR, 2.513; 95% CI, 2.056–3.072; *p* < 0.001), and after further adjustment for operative and tumor-related variables in Model 3 (OR, 2.568; 95% CI, 2.085–3.163; *p* < 0.001). The estimate remained stable after additional adjustment for surgical approach in Model 4 (OR, 2.587; 95% CI, 2.098–3.191; *p* < 0.001), conversion to open surgery in Model 5 (OR, 2.572; 95% CI, 2.086–3.171; *p* < 0.001), and both surgical approach and conversion in Model 6 (OR, 2.589; 95% CI, 2.097–3.196; *p* < 0.001).

### 3.9 Sensitivity, subgroup, and timing analyses

Sensitivity analyses using binary LAR categories were moved to Supplementary material to keep continuous LAR as the primary main-text exposure (Supplementary Table S2). Based on the ROC-derived cutoff of 5.855, postoperative pneumonia occurred in 24 of 235 patients (10.2%) in the low-LAR group and 123 of 232 patients (53.0%) in the high-LAR group. High LAR was associated with postoperative pneumonia in the crude model (OR, 9.921; 95% CI, 6.049–16.271; *p* < 0.001) and remained robust after staged adjustment. In Model 3, high LAR was associated with significantly higher odds of postoperative pneumonia (OR, 12.566; 95% CI, 7.287–21.670; *p* < 0.001), and the estimate remained stable in Model 6 after additional adjustment for surgical approach and conversion to open surgery (OR, 12.544; 95% CI, 7.269–21.647; *p* < 0.001). Prespecified subgroup analyses are shown in Supplementary Table S7 and Figure 4. The association between high LAR and postoperative pneumonia was observed across surgical approach, pathological stage, and FEV1/FVC strata. Formal interaction tests did not show statistically significant effect modification by surgical approach (P-interaction = 0.551), pathological stage (P-interaction = 0.354), or FEV1/FVC strata (P-interaction = 0.874). Sensitivity analyses for early and later postoperative pneumonia are shown in Supplementary Table S8 and Figure 2B. Compared with low LAR, high LAR was associated with early pneumonia occurring within POD 7 (adjusted OR, 12.465; 95% CI, 6.559–23.689; *p* < 0.001) and later pneumonia occurring from POD 8 to POD 30 (adjusted OR, 14.878; 95% CI, 5.996–36.918; *p* < 0.001).

### 3.10 Dose–response pattern and non-linearity

**Figure 8:**
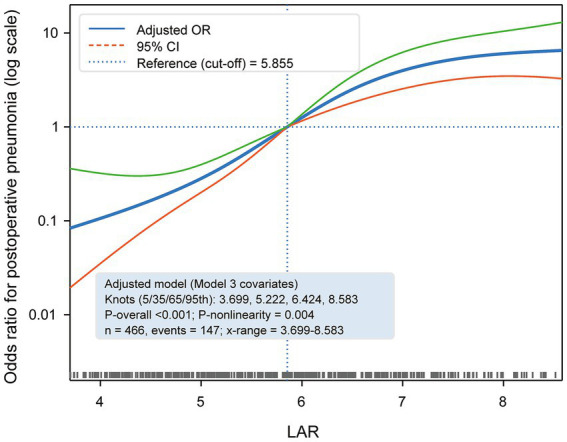
Restricted cubic spline analysis of the association between continuous preoperative LAR and postoperative pneumonia. The y-axis shows the adjusted odds ratio on a logarithmic scale, with the ROC-derived cutoff value of 5.855 used as the reference point. Knots were placed at the 5th, 35th, 65th, and 95th percentiles of the LAR distribution (3.699, 5.222, 6.424, and 8.583), and the x-axis was restricted to the 5th–95th percentile range. The model was adjusted using the Model 3 covariate set. The overall association was statistically significant (*P*-overall < 0.001), with evidence of non-linearity (*P*-non-linearity = 0.004).

Restricted cubic spline analysis was used to evaluate the functional form of the association between continuous LAR and postoperative pneumonia (Figures 8, 2A). With the ROC-derived cutoff of 5.855 used as the reference value and the x-axis restricted to the 5th–95th percentile range, the adjusted spline showed a significant overall association (P-overall < 0.001) and evidence of non-linearity (P-non-linearity = 0.004). RCS-derived risk strata are summarized in Supplementary Table S6 and Figure 2A. Pneumonia incidence increased from 13 of 164 patients (7.9%) in the low-risk stratum (LAR <5.222) to 36 of 139 patients (25.9%) in the intermediate stratum (LAR 5.222–6.424) and 98 of 164 patients (59.8%) in the high stratum (LAR >6.424). Compared with the low-risk stratum, the adjusted odds ratios were 4.980 (95% CI, 2.393–10.366; *p* < 0.001) for the intermediate stratum and 23.671 (95% CI, 11.627–48.191; *p* < 0.001) for the high stratum.

### 3.11 Clinical utility assessed by decision curve analysis

**Figure 9:**
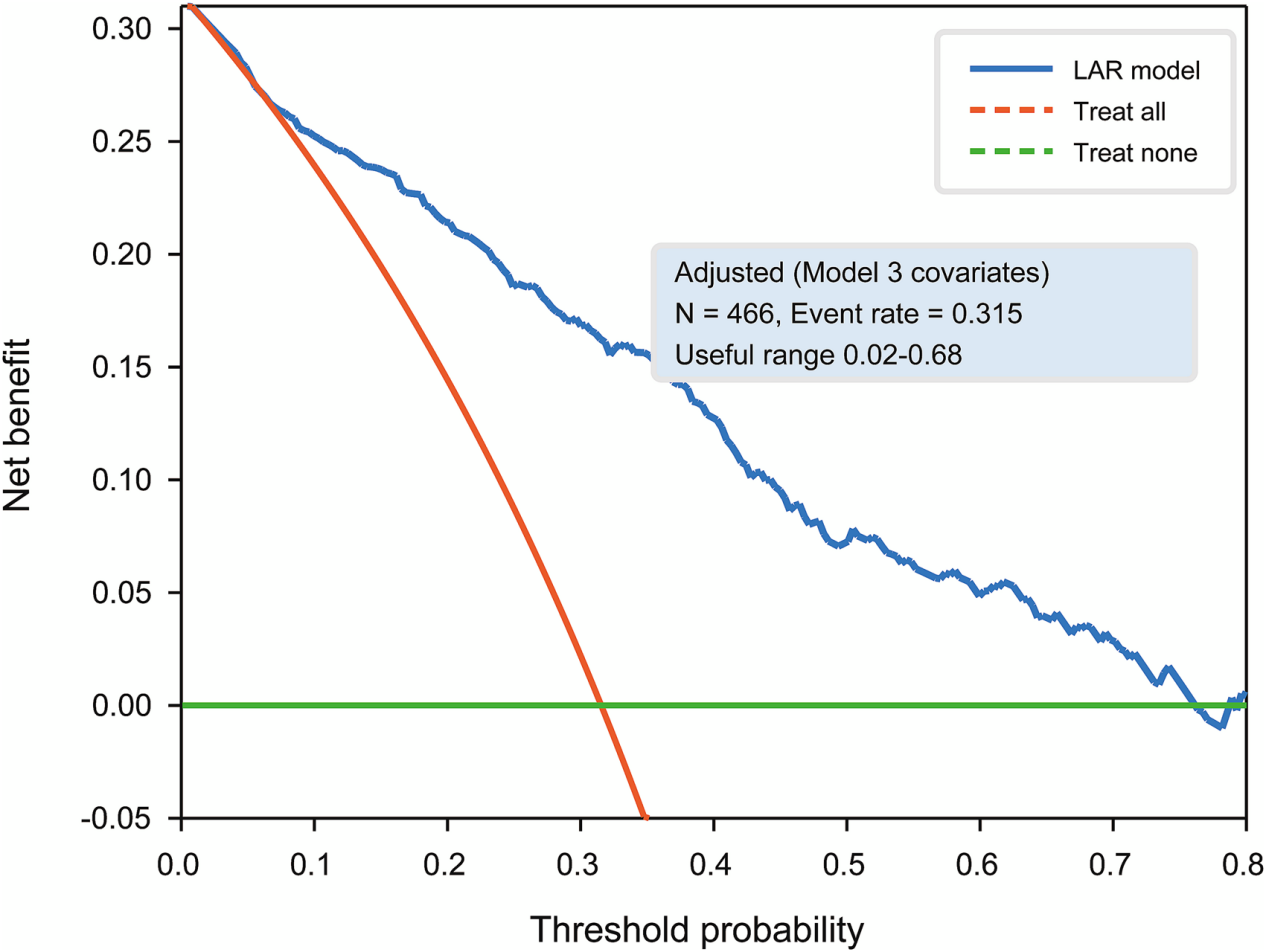
Decision curve analysis of the LAR-based adjusted model for postoperative pneumonia. Decision curve analysis was performed using predicted probabilities derived from the fully adjusted Model 3 with continuous LAR. Net benefit was compared across threshold probabilities against default treat-all and treat-none strategies. The model showed greater net benefit across threshold probabilities of 0.02–0.68, suggesting potential utility for perioperative risk stratification and individualized postoperative surveillance.

Decision curve analysis was performed to evaluate the clinical utility of the continuous LAR-based adjusted model (Figure 9). Using predicted probabilities derived from fully adjusted Model 3, the model showed greater net benefit than the treat-all and treat-none strategies across threshold probabilities of 0.02–0.68. These findings suggest that incorporating continuous LAR into an adjusted postoperative risk-stratification framework may provide clinical utility for identifying patients who may warrant closer postoperative surveillance and individualized perioperative optimization.

### 3.12 LDH–albumin interaction and joint-effect analyses

Exploratory LDH–albumin interaction and joint-effect analyses are shown in Supplementary Table S9 and Figure 5. Per one-standard-deviation increase, LDH was associated with higher odds of postoperative pneumonia (adjusted OR, 2.594; 95% CI, 1.912–3.519; *p* < 0.001). Lower albumin, modeled per one standard deviation decrease, was also associated with higher odds of pneumonia (adjusted OR, 3.637; 95% CI, 2.643–5.005; *p* < 0.001). The multiplicative interaction term was statistically significant but below 1.0 (adjusted OR, 0.726; 95% CI, 0.534–0.987; *p* = 0.041), indicating a non-additive relationship that should not be described as multiplicative synergy. In the joint-effect analysis, patients with low LDH and high albumin had the lowest pneumonia incidence (7/129, 5.4%) and were used as the reference group. Pneumonia incidence was higher among patients with high LDH only (27/105, 25.7%; adjusted OR, 6.505; 95% CI, 2.612–16.201), low albumin only (29/104, 27.9%; adjusted OR, 7.804; 95% CI, 3.149–19.341), and both high LDH and low albumin (84/129, 65.1%; adjusted OR, 38.289; 95% CI, 15.845–92.525). Exploratory additive interaction metrics suggested excess joint risk (RERI, 24.980; AP, 0.652; S, 3.029).

## 4 Discussion

In this single-center retrospective cohort of 467 consecutive patients undergoing curative-intent McKeown esophagectomy without neoadjuvant therapy, higher preoperative lactate dehydrogenase-to-albumin ratio (LAR) was consistently associated with postoperative pneumonia within 30 days after surgery. In the revised analysis framework, continuous LAR was retained as the primary exposure in the main text, whereas ROC-derived binary LAR was moved to Supplementary material as a sensitivity analysis. Continuous LAR showed good discrimination, remained independently associated with pneumonia after staged adjustment, and demonstrated a non-linear dose–response pattern. The association was further supported by subgroup, early versus later pneumonia, and LDH–albumin joint-effect analyses. These results indicate that LAR captures a clinically relevant preoperative nutrition–inflammation phenotype associated with postoperative pulmonary infectious morbidity after McKeown esophagectomy (24).

The cohort design should be interpreted in the context of its deliberate clinical restrictions. All patients underwent McKeown esophagectomy, and patients who received neoadjuvant chemotherapy, radiotherapy, immunotherapy, targeted therapy, or emergency surgery were excluded. This reduced heterogeneity from treatment-related immune, inflammatory, and nutritional perturbations. Patients with active preoperative infection were also excluded. Importantly, isolated elevation of CRP was not treated as an active infection by itself; rather, active infection required clinical evidence such as documented infectious diagnosis, antibiotic treatment for infection, compatible symptoms, microbiological evidence, or imaging findings. Therefore, this study did not exclude patients solely because of subclinical inflammatory burden, which is part of the biological construct that LAR and related markers may capture.

The decision to emphasize continuous LAR in the main analysis is important. A continuous biomarker model preserves information across the full exposure distribution and avoids overinterpreting a single data-derived threshold. In this cohort, each one-unit increase in LAR was associated with higher odds of postoperative pneumonia after full adjustment (Model 3 OR, 2.568; 95% CI, 2.085–3.163), and the estimate remained stable after additional adjustment for surgical approach and conversion to open surgery. The ROC-derived threshold of 5.855 was useful for clinical interpretability and sensitivity analysis, but it should be regarded as an internally derived benchmark rather than a universal decision threshold. External validation is required before this cutoff can be applied to other centers or perioperative pathways.

The comparative ROC analysis also requires a cautious interpretation. LAR had a higher AUC than albumin and LDH, and the difference between LAR and LDH was statistically significant. However, the difference between LAR and albumin did not reach statistical significance (*p* = 0.089). Therefore, LAR should not be presented as definitively superior to albumin alone in this dataset. A more defensible interpretation is that LAR provides a simple integrated index that combines LDH-related metabolic-inflammatory burden with albumin-related nutritional-inflammatory reserve. Albumin remained highly informative, while LAR may be most useful when clinicians want a composite signal rather than a replacement for individual laboratory measures (25).

The restricted cubic spline analysis strengthens the biological and clinical interpretation by showing that the association was not purely linear across the observed range. The overall association was significant, and there was evidence of non-linearity (P-non-linearity = 0.004). When patients were grouped according to spline-derived LAR strata, pneumonia incidence increased from 7.9% in the low-risk stratum to 25.9% in the intermediate stratum and 59.8% in the high-risk stratum. Compared with the low-risk stratum, the adjusted odds ratios were 4.980 for the intermediate stratum and 23.671 for the high stratum. These results support a graded risk pattern and provide clinically interpretable strata without making the binary ROC cutoff the central finding.

The subgroup and timing analyses, now presented as main figures, further address the robustness of the association. The association between high LAR and postoperative pneumonia was observed across surgical approach, pathological stage, and FEV1/FVC strata, and formal interaction tests did not show statistically significant effect modification. The timing analysis showed that the association was present for both early pneumonia occurring within POD 7 and later pneumonia occurring from POD 8 to POD 30. These findings suggest that elevated LAR is not merely a proxy for one surgical approach, tumor stage category, or baseline pulmonary function stratum. Nevertheless, subgroup analyses were exploratory, and some subgroups, especially hybrid and open surgery strata, had wide confidence intervals because of smaller sample sizes.

The perioperative descriptive analyses provide clinical context but should not be interpreted as causal predictors. Patients who developed postoperative pneumonia had higher rates of prolonged ventilation, reintubation, ICU readmission, parenteral nutrition use, delayed oral intake, delayed diet progression, higher postoperative CRP levels, longer SIRS duration, and longer fever duration. These variables are clinically coherent with pneumonia, but many can occur after pneumonia onset or be influenced by pneumonia itself. For this reason, they were summarized descriptively and were not included in the primary preoperative association models when they could represent downstream consequences of the outcome. This distinction is essential for avoiding overadjustment and for preventing postoperative consequences from being mislabeled as preoperative predictors.

The LDH–albumin analyses, now presented as a main figure, provide additional mechanistic context. Higher LDH and lower albumin were each associated with postoperative pneumonia when modeled per standard deviation. In the joint-effect analysis, patients with both high LDH and low albumin had the highest pneumonia incidence and the largest adjusted odds ratio compared with those with low LDH and high albumin. The multiplicative interaction term was statistically significant but below 1.0, so it should not be described as multiplicative synergy. A more appropriate interpretation is that LDH and albumin jointly identify a high-risk phenotype, while the exact scale and mechanism of interaction remain exploratory. The additive interaction metrics suggested excess joint risk, but these findings require validation.

Biologically, elevated LAR may reflect the convergence of LDH-related metabolic stress and albumin-related reduced host reserve. LDH may reflect tissue injury, hypoxia-related metabolic stress, anaerobic glycolysis, tumor–host metabolic activity, and systemic inflammation (15, 26). Albumin reflects nutritional reserve, but it is also a negative acute-phase reactant influenced by inflammation-related catabolism, vascular permeability, endothelial integrity, antioxidant capacity, and host defense (14, 16). In the setting of esophagectomy, a high-burden and low-reserve phenotype may reduce tolerance to surgical trauma, one-lung ventilation, aspiration exposure, postoperative pain-related cough impairment, and systemic inflammatory activation. This framework remains hypothesis-generating, and LAR should not be interpreted as a direct causal mediator of pneumonia.

It is also important to distinguish the present endpoint from long-term oncologic prognosis. Previous studies have evaluated LAR as a prognostic biomarker in cancer populations, including esophageal cancer (17, 18). Long-term survival is influenced by tumor biology, pathological stage, recurrence, adjuvant therapy, and competing risks. Postoperative pneumonia is an early perioperative event more directly related to the host’s inflammatory status, nutritional reserve, pulmonary vulnerability, aspiration risk, airway clearance, and surgical stress. In gastrointestinal cancer surgery, LAR has also been explored in relation to short-term outcomes and long-term prognosis (27). This study therefore extends the clinical context of LAR from long-term prognosis to short-term postoperative complication risk, without implying that the same threshold or effect size should be applied to survival prediction.

The timing findings have practical implications. Among patients who developed postoperative pneumonia, the median onset was POD 6, and events clustered most frequently during POD 4–7. For patients with elevated LAR, the most realistic clinical response is not automatic antibiotic therapy or delay of surgery based on LAR alone, but enhanced perioperative risk management. Potential actions include closer respiratory surveillance during the first postoperative week, structured pulmonary physiotherapy, cough and airway clearance support, aspiration precautions, analgesia optimization to facilitate deep breathing and coughing, early mobilization when feasible, careful fluid management, and individualized nutrition assessment (28, 29). These actions are consistent with ERAS recommendations and perioperative pulmonary-complication consensus guidance for esophagectomy (30, 31). Structured physiotherapy and airway clearance techniques have also been associated with fewer pulmonary complications after upper gastrointestinal surgery and esophagectomy (32, 33). Oral and dental optimization may also be relevant for reducing post-esophagectomy pulmonary complications (34). If LAR is identified earlier in the diagnostic or preoperative pathway, it may also support referral for nutritional and pulmonary prehabilitation, but a value measured within 7 days before surgery should mainly be used for risk recognition and postoperative care planning.

Traditional nutrition and frailty instruments remain important. We did not have systematic NRS2002, PG-SGA, sarcopenia, or frailty data for all patients, so this study cannot determine whether LAR adds incremental predictive value beyond validated nutrition or functional assessment tools. Recent studies have also linked prognostic nutritional index and geriatric nutritional risk index with postoperative pneumonia or postoperative complications in esophageal cancer populations (35, 36). This is a clinically relevant limitation rather than a reason to dismiss LAR. Because LDH and albumin are routinely available, LAR may be easy to calculate, but future studies should evaluate combined models incorporating LAR with NRS2002, PG-SGA, objective muscle measures, pulmonary function, and perioperative care variables.

This study has several strengths. First, the cohort was clinically homogeneous and restricted to patients undergoing McKeown esophagectomy without neoadjuvant therapy. Second, postoperative pneumonia was assessed within a prespecified 30-day window using radiographic evidence and objective clinical features. Third, the revised analysis used complementary approaches, including ROC analyses, staged multivariable models with continuous LAR, binary sensitivity analysis in the supplement, restricted cubic spline analyses, RCS-derived risk strata, decision curve analyses, subgroup analyses, early- and later-pneumonia analyses, and LDH–albumin joint-effect analyses. Fourth, the missing data structure was explicitly summarized, and complete-case denominators were reported for models affected by missing covariates.

Several limitations should be acknowledged. First, this was a single-center retrospective study, and residual confounding, selection bias, and center-specific perioperative practice patterns could not be excluded. Second, the ROC-derived cutoff and model performance were internally derived; external validation in independent multicenter cohorts is necessary before broader clinical generalization. Third, LAR was calculated from a single preoperative laboratory window within 7 days before surgery, and dynamic changes in LDH, albumin, CRP, and other inflammatory markers were not incorporated into the primary model. Fourth, although pneumonia was defined using radiographic and clinical criteria, retrospective outcome ascertainment may still be vulnerable to misclassification, particularly when atelectasis, aspiration, pulmonary edema, and infection overlap. Fifth, NRS2002, PG-SGA, sarcopenia, and frailty measures were not systematically available. Finally, this study did not evaluate long-term oncologic outcomes or test whether LAR-guided interventions improve postoperative outcomes.

In summary, preoperative LAR was independently associated with postoperative pneumonia after McKeown esophagectomy when analyzed primarily as a continuous nutrition–inflammation index. The association was non-linear, robust in binary sensitivity and subgroup analyses, and biologically supported by LDH–albumin joint-effect findings. LAR should be interpreted as a pragmatic risk-stratification signal rather than a stand-alone diagnostic, causal, or treatment-decision marker. Prospective multicenter validation is needed to confirm the optimal threshold, evaluate incremental value beyond established nutrition and functional assessments, and determine whether LAR-informed perioperative pathways can reduce postoperative pneumonia.

## Data Availability

The original contributions presented in the study are included in the article/Supplementary material, further inquiries can be directed to the corresponding authors.
