# Supplementary material for: Preoperative lactate dehydrogenase-to-albumin ratio as a nutrition–inflammation index associated with postoperative pneumonia after McKeown esophagectomy: a retrospective cohort study
Source: Front Nutr. 2026 Aug 5;13:1823767. doi: 10.3389/fnut.2026.1823767 (PMC13485821; doi:10.3389/fnut.2026.1823767)
Supplement: Supplementary file 1 [file Table_1.docx]

Supplementary Table S1. Univariable logistic regression analyses for postoperative pneumonia.

| Variable | n | Events | OR (95% CI) | P value |
| --- | --- | --- | --- | --- |
| Age (years) [per 1 year] | 467 | 147 | 0.998 (0.969-1.028) | 0.891 |
| Sex | 467 | 147 |  | 0.210 |
| Female |  |  | Ref |  |
| Male |  |  | 0.778 (0.526-1.152) | 0.210 |
| BMI (kg/m2) [per 1 kg/m2] | 467 | 147 | 0.993 (0.914-1.079) | 0.863 |
| Smoking status | 467 | 147 |  | 0.298 |
| No |  |  | Ref |  |
| Yes |  |  | 1.242 (0.826-1.867) | 0.298 |
| Alcohol consumption | 467 | 147 |  | 0.558 |
| No |  |  | Ref |  |
| Yes |  |  | 1.133 (0.746-1.719) | 0.558 |
| Hypertension | 467 | 147 |  | 0.702 |
| No |  |  | Ref |  |
| Yes |  |  | 1.079 (0.730-1.595) | 0.702 |
| Diabetes mellitus | 467 | 147 |  | 0.995 |
| No |  |  | Ref |  |
| Yes |  |  | 1.002 (0.585-1.715) | 0.995 |
| Coronary heart disease | 467 | 147 |  | 0.481 |
| No |  |  | Ref |  |
| Yes |  |  | 1.157 (0.771-1.737) | 0.481 |
| ASA physical status | 467 | 147 |  | 0.644 |
| I |  |  | Ref |  |
| II |  |  | 1.185 (0.766-1.834) | 0.445 |
| III |  |  | 1.547 (0.477-5.015) | 0.467 |
| FEV1/FVC (%) [per 1%] | 467 | 147 | 1.013 (0.996-1.030) | 0.125 |
| LVEF (%) [per 1%] | 467 | 147 | 1.045 (0.958-1.139) | 0.324 |
| Albumin (g/L) [per 1 g/L] | 467 | 147 | 0.765 (0.719-0.815) | <0.001 |
| LDH (U/L) [per 1 U/L] | 467 | 147 | 1.019 (1.013-1.024) | <0.001 |
| LAR [per 1 unit] | 467 | 147 | 2.389 (1.976-2.890) | <0.001 |
| Surgical approach | 467 | 147 |  | 0.678 |
| MIE McKeown |  |  | Ref |  |
| Hybrid McKeown |  |  | 1.068 (0.585-1.951) | 0.831 |
| Open McKeown |  |  | 1.353 (0.687-2.662) | 0.382 |
| Conversion to open | 467 | 147 |  | 0.357 |
| No |  |  | Ref |  |
| Yes |  |  | 1.660 (0.565-4.872) | 0.357 |
| Operative duration (min) [per 1 min] | 467 | 147 | 1.006 (1.001-1.011) | 0.011 |
| Single-lung ventilation time (min) [per 1 min] | 467 | 147 | 1.000 (0.996-1.005) | 0.832 |
| Intraoperative blood loss (mL) [per 1 mL] | 467 | 147 | 1.002 (0.999-1.005) | 0.264 |
| RLN injury | 467 | 147 |  | 0.291 |
| No |  |  | Ref |  |
| Yes |  |  | 0.606 (0.239-1.534) | 0.291 |
| Tumor location | 467 | 147 |  | 0.069 |
| Upper |  |  | Ref |  |
| Middle |  |  | 1.003 (0.611-1.646) | 0.991 |
| Lower |  |  | 1.665 (0.969-2.862) | 0.065 |
| Tumor length (cm) [per 1 cm] | 466 | 147 | 0.882 (0.664-1.172) | 0.387 |
| Tumor differentiation | 467 | 147 |  | 0.546 |
| Well |  |  | Ref |  |
| Moderate |  |  | 0.803 (0.340-1.896) | 0.617 |
| Poor |  |  | 1.006 (0.433-2.336) | 0.989 |
| pT category | 467 | 147 |  | 0.963 |
| T1 |  |  | Ref |  |
| T2 |  |  | 1.059 (0.681-1.647) | 0.800 |
| T3 |  |  | 1.059 (0.613-1.830) | 0.836 |
| pN category | 467 | 147 |  | 0.984 |
| N0 |  |  | Ref |  |
| N1 |  |  | 1.004 (0.678-1.487) | 0.984 |
| pTNM stage | 467 | 147 |  | 0.881 |
| I |  |  | Ref |  |
| II |  |  | 1.118 (0.718-1.742) | 0.622 |
| III |  |  | 1.094 (0.631-1.896) | 0.749 |

*Footnote. Univariable logistic regression was performed to evaluate associations with postoperative pneumonia. ORs are reported per 1-unit increase for continuous variables and relative to the specified reference category for categorical variables.*

Supplementary Table S2. Sensitivity analyses using binary LAR categories for postoperative pneumonia.

| Model | n | Events | OR (95% CI) for high vs low LAR | P value |
| --- | --- | --- | --- | --- |
| Crude | 467 | 147 | 9.921 (6.049-16.271) | <0.001 |
| Model 1 | 467 | 147 | 10.183 (6.183-16.771) | <0.001 |
| Model 2 | 467 | 147 | 11.049 (6.597-18.507) | <0.001 |
| Model 3 | 466 | 147 | 12.566 (7.287-21.670) | <0.001 |
| Model 4 | 466 | 147 | 12.576 (7.289-21.697) | <0.001 |
| Model 5 | 466 | 147 | 12.535 (7.268-21.618) | <0.001 |
| Model 6 | 466 | 147 | 12.544 (7.269-21.647) | <0.001 |

*Footnote. Binary LAR categories were defined using the ROC-derived Youden-index cut-off of 5.855; high LAR was defined as LAR >= 5.855 and low LAR as LAR < 5.855. ORs compare high vs low LAR using the same staged adjustment strategy as the continuous LAR models.*

Supplementary Table S3. Timing of postoperative pneumonia onset according to LAR category.

| Characteristic | Overall \| (N=467) | Low LAR \| (<5.855; n=235) | High LAR \| (>=5.855; n=232) | P value |
| --- | --- | --- | --- | --- |
| Postoperative pneumonia status in the full cohort |  |  |  | <0.001 |
| No postoperative pneumonia | 320 (68.5%) | 211 (89.8%) | 109 (47.0%) |  |
| Postoperative pneumonia | 147 (31.5%) | 24 (10.2%) | 123 (53.0%) |  |
| Timing category in the full cohort |  |  |  | <0.001 |
| No postoperative pneumonia | 320 (68.5%) | 211 (89.8%) | 109 (47.0%) |  |
| Early pneumonia (POD <=7) | 98 (21.0%) | 17 (7.2%) | 81 (34.9%) |  |
| Later pneumonia (POD 8-30) | 49 (10.5%) | 7 (3.0%) | 42 (18.1%) |  |
| Timing of pneumonia onset among pneumonia cases |  |  |  |  |
| Pneumonia cases included, n | 147 | 24 | 123 |  |
| Onset POD, median (IQR) | 6.0 (4.0-9.0) | 5.0 (4.0-9.0) | 6.0 (4.0-9.0) | 0.901 |
| Onset period among pneumonia cases |  |  |  | 0.300 |
| POD 1-3 | 30 (20.4%) | 4 (16.7%) | 26 (21.1%) |  |
| POD 4-7 | 68 (46.3%) | 13 (54.2%) | 55 (44.7%) |  |
| POD 8-14 | 40 (27.2%) | 4 (16.7%) | 36 (29.3%) |  |
| POD 15-30 | 9 (6.1%) | 3 (12.5%) | 6 (4.9%) |  |

*Footnote. In the full-cohort sections, percentages were calculated using the total number of patients in each LAR category as the denominator. In the pneumonia-case sections, percentages were calculated using the number of patients with postoperative pneumonia in each LAR category as the denominator. The P value for postoperative pneumonia status and timing category was obtained using the chi-square test. The P value for onset POD was obtained using the Mann-Whitney U test; the P value for onset-period distribution among pneumonia cases was obtained using the chi-square test. Early pneumonia was defined as pneumonia occurring on or before postoperative day 7.*

Supplementary Table S4. Postoperative outcomes and inflammatory response according to LAR category.

| Characteristic | Overall \| (n=467) | Low LAR \| (<5.855; n=235) | High LAR \| (>=5.855; n=232) | P value | \|SMD\| |
| --- | --- | --- | --- | --- | --- |
| Postoperative pulmonary and ICU outcomes |  |  |  |  |  |
| Postoperative pneumonia, yes | 147 (31.5%) | 24 (10.2%) | 123 (53.0%) | <0.001 | 1.037 |
| Prolonged ventilation >=24 h, yes | 37 (7.9%) | 10 (4.3%) | 27 (11.6%) | 0.003 | 0.276 |
| Reintubation, yes | 17 (3.6%) | 2 (0.9%) | 15 (6.5%) | 0.001 | 0.302 |
| ICU readmission, yes | 27 (5.8%) | 6 (2.6%) | 21 (9.1%) | 0.003 | 0.281 |
| Cumulative ICU stay (days) | 1.0 (1.0-2.0) | 1.0 (1.0-2.0) | 1.0 (1.0-2.0) | 0.039 | 0.258 |
| Postoperative nutritional recovery and mobilization |  |  |  |  |  |
| Parenteral nutrition, yes | 63 (13.5%) | 21 (8.9%) | 42 (18.1%) | 0.004 | 0.271 |
| Oral liquid start (POD) | 7.0 (7.0-9.0) | 7.0 (6.0-8.0) | 8.0 (7.0-9.2) | <0.001 | 0.460 |
| Soft diet start (POD) | 11.0 (10.0-13.0) | 11.0 (10.0-12.0) | 12.0 (10.0-14.0) | <0.001 | 0.392 |
| Ambulation within POD1, yes | 253 (54.2%) | 144 (61.3%) | 109 (47.0%) | 0.002 | 0.290 |
| Perioperative inflammatory response |  |  |  |  |  |
| Preoperative CRP (mg/L) | 4.7 (3.5-6.3) | 4.1 (3.3-5.4) | 5.4 (4.0-7.0) | <0.001 | 0.596 |
| CRP on POD3 (mg/L) | 130.3 (110.8-153.4) | 127.0 (109.9-147.5) | 135.6 (113.5-161.1) | 0.011 | 0.260 |
| CRP on POD5 (mg/L) | 90.1 (69.2-120.0) | 81.3 (62.9-104.7) | 101.4 (75.9-132.3) | <0.001 | 0.562 |
| CRP on POD7 (mg/L) | 63.3 (41.6-89.2) | 52.8 (38.2-72.8) | 72.2 (49.6-107.0) | <0.001 | 0.621 |
| Peak CRP on POD0-7 (mg/L) | 137.6 (118.2-159.5) | 129.5 (115.3-151.0) | 143.5 (120.6-170.2) | <0.001 | 0.444 |
| SIRS duration on POD0-30 (days) | 1.0 (0.0-2.0) | 1.0 (0.0-2.0) | 2.0 (0.8-3.0) | <0.001 | 0.474 |
| SIRS >=48 h, yes | 196 (42.0%) | 69 (29.4%) | 127 (54.7%) | <0.001 | 0.532 |
| Fever duration (days) | 1.0 (0.0-2.0) | 1.0 (0.0-2.0) | 2.0 (0.0-3.0) | <0.001 | 0.469 |

*Footnote. Data are presented as n (%) or median (IQR), as appropriate. Low LAR was defined as LAR <5.855 and high LAR as LAR >=5.855 according to the ROC-derived Youden index cut-off. Skewed continuous variables were compared using the Mann-Whitney U test. Categorical variables were compared using the chi-square test or Fisher's exact test, as appropriate. Absolute standardized mean differences (|SMD|) were calculated to quantify between-group imbalance.*

Supplementary Table S5. Missing data summary.

| Variable | Missing n | Missing % | No pneumonia missing n | Pneumonia missing n | Note |
| --- | --- | --- | --- | --- | --- |
| Tumor_length | 1 | 0.2 | 1 | 0 | Only missing value among Model 3 covariates; adjusted complete-case models use n=466. |
| Pneumonia_onset_POD | 320 | 68.5 | 320 | 0 | Structurally missing among patients without postoperative pneumonia. |
| Early_pneumonia_le7d | 320 | 68.5 | 320 | 0 | Structurally missing among patients without postoperative pneumonia. |
| Enteral_nutrition_start_POD | 10 | 2.1 | 7 | 3 | Postoperative descriptive variable; not used in primary preoperative prediction models. |
| Early_vs_none | 49 | 10.5 | 0 | 49 |  |
| Late_vs_none | 98 | 21.0 | 0 | 98 |  |

*Footnote. Pneumonia timing variables were structurally missing among patients without postoperative pneumonia. Tumor_length had one missing value; adjusted complete-case models therefore used n=466.*

Supplementary Table S6. Pneumonia incidence and odds ratios across RCS-derived LAR risk strata.

| LAR risk stratum | N | Events | Incidence (%) | Crude OR (95% CI) | P | Adjusted OR (95% CI) | P |
| --- | --- | --- | --- | --- | --- | --- | --- |
| Low (<5.222) | 164 | 13 | 7.9 | Reference |  | Reference |  |
| Intermediate (5.222-6.424) | 139 | 36 | 25.9 | 4.060 (2.053-8.029) | <0.001 | 4.980 (2.393-10.366) | <0.001 |
| High (>6.424) | 164 | 98 | 59.8 | 17.247 (9.033-32.932) | <0.001 | 23.671 (11.627-48.191) | <0.001 |

*Footnote. Risk strata were defined using the internal RCS knots at LAR 5.222 and 6.424. Adjusted ORs used Model 3 covariates.*

Supplementary Table S7. Subgroup analyses for the association between high LAR and postoperative pneumonia.

| Subgroup family | Level | N | Events | Incidence (%) | Adjusted OR (95% CI) | P | P interaction |
| --- | --- | --- | --- | --- | --- | --- | --- |
| Surgical approach | MIE McKeown | 371 | 114 | 30.7 | 10.030 (5.725-17.573) | <0.001 | 0.551 |
| Surgical approach | Hybrid McKeown | 56 | 18 | 32.1 | 24.397 (3.912-152.158) | <0.001 | 0.551 |
| Surgical approach | Open McKeown | 40 | 15 | 37.5 | 5.091 (1.014-25.556) | 0.048 | 0.551 |
| Surgical approach, two-group | MIE | 371 | 114 | 30.7 | 10.030 (5.725-17.573) | <0.001 | 0.905 |
| Surgical approach, two-group | Hybrid/Open | 96 | 33 | 34.4 | 11.670 (3.767-36.152) | <0.001 | 0.905 |
| Pathological stage | I | 160 | 48 | 30.0 | 16.417 (5.906-45.638) | <0.001 | 0.354 |
| Pathological stage | II | 213 | 69 | 32.4 | 7.282 (3.645-14.549) | <0.001 | 0.354 |
| Pathological stage | III | 94 | 30 | 31.9 | 14.181 (4.375-45.970) | <0.001 | 0.354 |
| FEV1/FVC | <median FEV1/FVC | 223 | 61 | 27.4 | 10.530 (4.676-23.715) | <0.001 | 0.874 |
| FEV1/FVC | >=median FEV1/FVC | 244 | 86 | 35.2 | 11.808 (6.055-23.026) | <0.001 | 0.874 |

*Footnote. Adjusted ORs compare high vs low LAR within each subgroup and were adjusted for age, sex, and BMI. P interaction indicates the interaction P value within each subgroup family.*

Supplementary Table S8. Sensitivity analyses for early and later postoperative pneumonia.

| Outcome | N | Events | Incidence (%) | Crude OR (95% CI) | P | Adjusted OR (95% CI) | P |
| --- | --- | --- | --- | --- | --- | --- | --- |
| Any 30-day postoperative pneumonia | 467 | 147 | 31.5 | 9.921 (6.049-16.271) | <0.001 | 12.566 (7.287-21.670) | <0.001 |
| Early pneumonia <=7 days vs no pneumonia | 418 | 98 | 23.4 | 9.223 (5.207-16.337) | <0.001 | 12.465 (6.559-23.689) | <0.001 |
| Later pneumonia >7 days vs no pneumonia | 369 | 49 | 13.3 | 11.615 (5.050-26.713) | <0.001 | 14.878 (5.996-36.918) | <0.001 |

*Footnote. Early pneumonia was defined as onset on or before POD 7; later pneumonia was defined as POD 8-30. ORs compare high vs low LAR.*

Supplementary Table S9. LDH-albumin interaction and joint-effect analyses.

| Analysis type | Term/group | N | Events | Incidence (%) | Estimate (95% CI) | P | Note |
| --- | --- | --- | --- | --- | --- | --- | --- |
| Continuous model | LDH per 1 SD increase | 466 | 147 |  | 2.594 (1.912-3.519) | <0.001 |  |
| Continuous model | Lower albumin per 1 SD decrease | 466 | 147 |  | 3.637 (2.643-5.005) | <0.001 |  |
| Continuous model | LDH x lower albumin interaction | 466 | 147 |  | 0.726 (0.534-0.987) | 0.041 |  |
| Joint categories | Low LDH / high albumin | 129 | 7 | 5.4 | Reference |  |  |
| Joint categories | High LDH only | 105 | 27 | 25.7 | 6.505 (2.612-16.201) | <0.001 |  |
| Joint categories | Low albumin only | 104 | 29 | 27.9 | 7.804 (3.149-19.341) | <0.001 |  |
| Joint categories | High LDH / low albumin | 129 | 84 | 65.1 | 38.289 (15.845-92.525) | <0.001 |  |
| Additive metrics | Additive interaction: RERI | 466 | 147 |  | 24.980 |  | RERI >0 suggests positive additive interaction; no bootstrap CI computed. |
| Additive metrics | Additive interaction: AP | 466 | 147 |  | 0.652 |  | Attributable proportion due to additive interaction; descriptive only. |
| Additive metrics | Additive interaction: S index | 466 | 147 |  | 3.029 |  | Synergy index on additive scale; descriptive only. |

*Footnote. LDH and albumin were standardized in the continuous interaction model. Joint categories used cohort medians. Adjusted estimates used Model 3 covariates.*
